# Analysis of purified Wild type and mutant adenovirus particles by SILAC based quantitative proteomics

**DOI:** 10.1099/vir.0.068221-0

**Published:** 2014-11

**Authors:** Ali Alqahtani, Kate Heesom, Jonathan L. Bramson, David Curiel, Hideyo Ugai, David A. Matthews

**Affiliations:** 1 School of Cellular and Molecular Medicine, University Walk, University of Bristol, Bristol BS8 1TD, UK; 2 College of Applied Medical Sciences, Najran University, Najran 1983, Saudi Arabia; 3 Proteomics Facility, Faculty of Medical and Veterinary Sciences, University Walk, University of Bristol, Bristol BS8 1TD, UK; 4 McMaster Immunology Research Centre, 4016 Michael DeGroote Centre for Learning & Discovery, McMaster University, Hamilton, L8S 4L8 Ontario, Canada; 5 Cancer Biology Division, Department of Radiation Oncology, School of Medicine, Washington University in St Louis, 4511 Forest Park Medical Building, St Louis, MO 63108, USA; 6 Biologic Therapeutics Center, School of Medicine, Washington University in St Louis, 4511 Forest Park Medical Building, St Louis, MO 63108, USA

## Abstract

We used SILAC (stable isotope labelling of amino acids in cell culture) and high-throughput quantitative MS mass spectrometry to analyse the protein composition of highly purified WT wild type adenoviruses, mutant adenoviruses lacking an internal protein component (protein V) and recombinant adenoviruses of the type commonly used in gene therapy, including one virus that had been used in a clinical trial. We found that the viral protein abundance and composition were consistent across all types of virus examined except for the virus lacking protein V, which also had reduced amounts of another viral core protein, protein VII. In all the samples analysed we found no evidence of consistent packaging or contamination with cellular proteins. We believe this technique is a powerful method to analyse the protein composition of this important gene therapy vector and genetically engineered or synthetic virus-like particles. The raw data have been deposited at proteomexchange, identifer PXD001120.

Adenoviruses are non-enveloped, icosahedral and enclose a dsDNA genome of ~36 000 bp (Shenk, 1996). The major components of the capsid are the proteins hexon, penton base and fibre (proteins II, III and IV, respectively); the capsid is also stabilized by four minor proteins IIIa, VI, VIII and IX. The virus core contains another six proteins; three are non-covalently associated with the viral genome (proteins V, VII and X), the terminal protein (TP) is covalently attached to the 5′ ends of the viral genome and two more proteins are present in the capsid (the 23K viral protease and protein IVa2) (Chatterjee *et al.*, 1985; Everitt *et al.*, 1973; Giberson *et al.*, 2012; Nemerow *et al.*, 2012; Russell, 2009; San Martín, 2012; van Oostrum & Burnett, 1985; Vellinga *et al.*, 2005). Assembly of adenovirus particles occurs in the infected cell nucleus through four different stages: light intermediate (empty particle), heavy intermediate (incomplete particle), young virion and mature virion (Edvardsson *et al.*, 1976; Ishibashi & Maizel, 1974). Empty and incomplete particles have no DNA or associated core proteins (e.g. V, VII and X), whilst young and mature virions do (Ishibashi & Maizel, 1974).

Proteomic analysis of adenovirus particles by tandem MS (MS/MS) has identified peptides for most of the adenovirus structural proteins along with some phosphorylation sites (Benevento *et al.*, 2014; Bergström Lind *et al.*, 2012; Chelius *et al.*, 2002; Liu *et al.*, 2003). MS/MS has also been used to investigate the protein composition of empty adenovirus particles compared with mature virus particles or heat-disrupted particles (Benevento *et al.*, 2014; Takahashi *et al.*, 2006). The human SET and nucleolin proteins were identified by this approach as common contaminants in purified preparations of adenovirus particles (Riske *et al.*, 2013). However, none of these studies analysed multiple batches of purified virus to examine the consistency of the proteomic composition of the virus. For example, we do not formally know if purified virus composition is affected by changing the cell line or laboratory purifying the particles. We do not know which cellular proteins are random contaminants or are associated consistently with the virus. Moreover, we have no data on the proteomic composition of recombinant adenoviruses with deletions in the viral genome that may lead to reduced viral DNA-packaging proteins. Finally, if viral structural proteins are altered, reduced or deleted, this may allow the packaging of other viral or cellular proteins. A quantitative proteomics-based analysis allows several batches to be compared to determine the relative abundance of detected proteins. Only proteins present normally in the virus preparation will have a consistent relative abundance ratio of ~1 : 1.

This has utility for the analysis of batch-to-batch variation, novel mutant virus or virus-like particle (VLP) composition and even biosynthetic nanoparticle composition. For example, adenoviruses have been modified structurally to make therapeutic virus or useful VLPs to deliver therapeutic agents (Naskalska *et al.*, 2013). Moreover, we have worked on a mutant adenovirus called dV/TSB, lacking the structural viral core protein V and with point mutations in the precursor region of structural protein X (Ugai *et al.*, 2007). Interestingly, dV/TSB has an assembly defect in primary cells, but not in cancer cells (Ugai *et al.*, 2012). Whether the removal of protein V creates physical space for other proteins to occupy or if this affects the packaging of other viral proteins is unknown.

To quantitatively analyse adenovirus particles, we used MS coupled with SILAC (stable isotope labelling of amino acids in cell culture), as described previously (Evans *et al.*, 2012). We investigated the protein composition of dV/TSB and compared it with WT human adenovirus type 5 (Ad5 strain Ad75), an Ad5 based E1- and E3-deleted adenovirus expressing EGFP from a cytomegalovirus promoter (produced using the AdMAx system; Microbix), and a batch of a similar recombinant adenovirus being developed as a booster vaccine for tuberculosis, which has been used in a clinical trial (Smaill *et al.*, 2013). Adenoviruses were propagated in A549 cells, 293 cells or HeLa cells labelled with either no stable isotopes, or R6K6 or R10K8 isotope combinations (Table S1, available in the online Supplementary Material). Each batch of virus from the infected cells was purified twice by using a caesium chloride gradient (Kanegae *et al.*, 1994; Ugai *et al.*, 2005) (Fig. S1).

Each batch of virus contained only the major viral proteins normally detected by SDS-PAGE and Coomassie staining (Fig. 1a–c). We found that protein V was missing from all dV/TSB preparations (Fig. 1b), and proteins V and VII were missing from immature particles in WT Ad5 and dV/TSB, as expected (Fig. 1c).

**Fig. 1.**
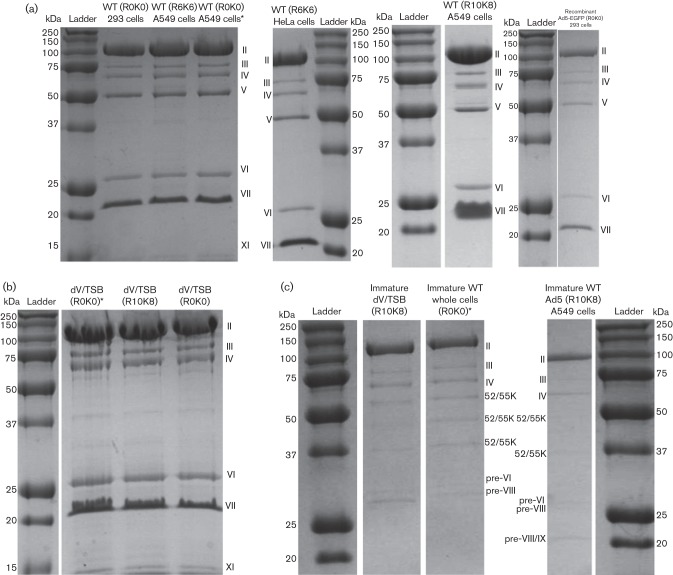

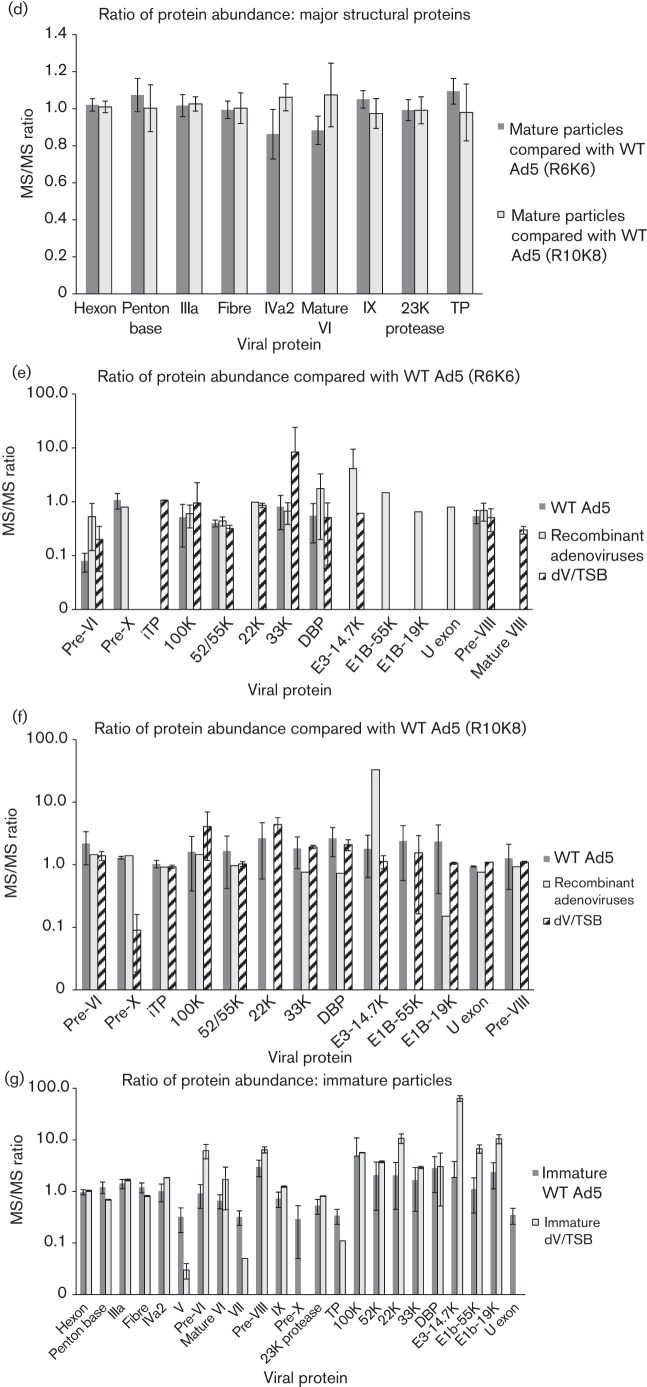
Abundance ratios of viral proteins detected and quantified. Single batches of WT adenovirus labelled with R6K6 or R10K8 were compared quantitatively with 11 or six different virus batches, respectively. (a–c) Coomassie-stained gels of the viruses. The type of virus used, whether mature or immature particles and isotopic labelling are indicated. Samples marked with an asterisk were purified by H. U. at Washington State University at St Louis. (a) Mature WT particles and mature recombinant adenoviruses, (b) mature dV/TSB particles, and (c) immature particles. For each gel, a ladder with molecular mass standards is included and the virus proteins are labelled. (d–g) Mean ratio±sd (two or more values available). Those viral proteins where the ratio of abundance was ~1 : 1 in all experiments are shown in (d). Note that all subsequent charts use a log scale because of the wide variability of abundance. (e) Range of quantification using R6K6 virus as the comparator and (f) the same proteins where their abundance ratios were compared with the R10K8 virus preparation. (g) A similar analysis of immature particles where the relative abundance of viral proteins in immature particles was compared with the batch of R6K6-labelled mature particles used in (d, e).

We compared batches prepared at the University of Bristol and at Washington University in St Louis, and one sample of recombinant adenovirus Ag85A used recently in a clinical trial (Smaill *et al.*, 2013). Comparing purified viruses derived from distinct cell lines, purified by different individuals in different laboratories, reduces the potential for systematic artefactual contamination in one laboratory or with a particular cell line. Twelve different mixtures of these virus preparations (Table S1) were analysed by high-throughput quantitative MS/MS, as described previously (Evans *et al.*, 2012). The MS/MS spectral data were searched using MaxQuant (Cox *et al.*, 2011) against a database of human proteins derived from UniProt, but with additional adenovirus protein sequences (provided with the PRIDE dataset submission; http://www.ebi.ac.uk/pride/archive/). Some adenovirus structural proteins are made as precursor proteins that are proteolytically matured by the virus-coded 23K proteinase. Thus, protein entries corresponding to the mature, processed viral proteins need to be included in the protein database.

We detected a mean of 556 peptides in each mixture of viruses (Table 1) and identified a total of 27 viral proteins covered by two or more distinct peptides, and detected all major and most minor structural viral proteins. The major structural proteins [hexon (II), penton base (III) and fibre (IV)] as well as some minor structural proteins (pre-IIIa, IIIa, IVa2, IX, 23K protease, pre-TP and TP) were identified and quantified as being present in a similar ratio (~1 : 1) between different batches of mature particles (Fig. 1d). Inexplicably, we were unable to reliably detect peptides unique to intermediate TP (iTP) in all our samples. We also noted that the immature particles had a similar 1 : 1 ratio as the major structural proteins (II, III and IV) (Fig. 1g). Consistent ratios of major structural proteins between various samples from three different cell lines and three different laboratories on two continents provided reassurance about the accuracy and reproducibility of this kind of analysis. Moreover, normally the TP and the 23K protease are present at only two and 10 copies per virus, respectively (Rekosh, 1981; Stewart & Burnett, 1995). Despite their low abundance, both proteins were detected at ~1 : 1 ratio in all of the virus preparations (Fig. 1d). This indicated that any protein present consistently in the virus particle could, in principle, be detected and quantified even if there were only two copies per virion.

**Table 1.** Identification of viral proteins from several viruses labelled with different isotopes This table lists, for each virus protein detected, in how many samples the protein was detected and how many peptides were identified in each of the 12 different analyses of virus particles.

Viral protein	No. of times protein identified in MS/MS analysis (maximum of 19 analyses)	Mean no. of peptides identified in each analysis	Mean sequence coverage (%)
Hexon (II)	19	105	83.51
Penton base (III)	19	39	66.77
Pre-IIIa and mature IIIa	19	52	81.56
Fibre (IV)	19	26	57.01
IVa2	19	27	62.13
V	19	35	55.93
Pre-VI	19	15	77.70
Mature VI	19	19	84.10
Pre-VII and mature VII	19	22	64.13
Pre-VIII and mature VIII	10	14	74.90
Pre-VIII	17	12	64.92
Mature VIII	17	6	81.26
IX	19	11	86.97
X	15	2	18.89
23K protease	19	18	57.53
TP and pre-TP	19	18	28.93
iTP	3	5	36.63
Late 100K	19	37	48.33
52/55K	19	33	62.61
22K	10	6	38.21
33K	19	7	34.97
DBP	19	18	38.98
E3-14.7K	13	4	26.77
E1B-55K	9	6	13.49
E1B-19K	13	8	50.74
U exon	11	7	24.40
E3-12.5K	5	2	18.25
E4-14.7K	5	2	10.00

We compared our data on the mature particles with that of previous studies (Bergström Lind *et al.*, 2012; Chelius *et al.*, 2002). We found that some minor viral proteins that are known to be part of mature particles (pre-X and IVa2) (D’Halluin *et al.*, 1978; Takahashi *et al.*, 2006) as well as several minor viral proteins believed to be part of immature particles [pre-VI, 100K, 22K, 33K, DNA-binding protein (DBP), E3-14.7K, U exon, E1B-19K and E1B-55K] (Boudin *et al.*, 1980; D’Halluin *et al.*, 1978; Edvardsson *et al.*, 1976; Pérez-Berná *et al.*, 2009; Sutjipto *et al.*, 2005; Takahashi *et al.*, 2006) were detected for the first time using LC-MS/MS. We believe that the detection of proteins not thought to be bona fide components of mature particles reflected minor contamination of the mature particles with immature particles (as is common) and this would explain the observation that their abundance ratios were inconsistent.

Turning to the analysis of immature particles, they show greatly reduced amounts of proteins V, VII and TP compared with mature particles (Fig. 1g). This is in line with previous studies showing that proteins V and VII are not present in the immature particles, and is consistent with the lack of DNA (Everitt *et al.*, 1973; Ishibashi & Maizel, 1974; Sundquist *et al.*, 1973). Some non-structural proteins (100K and DBP) were found with a similar elevated ratio of abundance to mature WT particles in all types of immature particles (Fig. 1e, g). The data confirm that these non-structural proteins are depleted in mature particles.

As expected, protein V was present in similar amounts in mature WT Ad5 and both recombinant adenoviruses. Moreover, proteins pre-VII and mature VII were present at a notably reduced amount in mature dV/TSB as compared with the mature WT Ad5 and both recombinant adenoviruses (Fig. 2a). This apparent reduction in protein VII was not noticed in our initial inspection of the Coomassie-stained gels used to assess the purity of each preparation. Thus, to assess this discrepancy further, Ad5 and dV/TSB were re-examined carefully by SDS-PAGE and Coomassie blue staining. This showed that WT Ad5 and dV/TSB contained similar amounts of all the major structural proteins except for protein VII, which was less abundant in dV/TSB (Fig. 2b). That there was less protein VII is not related to the size of the dV/TSB genome as the recombinant adenoviruses are deleted for regions E1 and E3, and will have smaller genomes than dV/TSB. The reduced level of protein VII is likely related to the lack of protein V or because in dV/TSB the precursor to X (pre-X) contains missense mutations (Ugai *et al.*, 2007). Proteins V, VII and X are known to play roles in packaging the viral genome (Russell, 2009), with mature proteins VII and X being tightly associated with the viral DNA (Black & Center, 1979). Furthermore, direct interaction between proteins V, VII and X has been detected (Chatterjee *et al.*, 1985). As the dV/TSB DNA could be packaged efficiently with reduced levels of protein VII and no protein V, we infer that there are significant gaps in our understanding of how adenovirus packages its DNA. We speculate that not all the protein VII is needed to neutralize/package viral DNA and a proportion of protein VII is not associated directly with the viral DNA. Wang *et al.* (2013) reported significant amounts of protein VII dissociating from viral DNA on entry to the cell, which may support our speculation. Notably, we did not detect pre-X in dV/TSB by MS/MS, whilst WT Ad5 and both recombinant adenoviruses had an almost 1 : 1 ratio of peptides from pre-X. The original publication describing dV/TSB examined the composition of dV/TSB by silver stained SDS-PAGE gels, which indicated that there may be more mature protein X in the dV/TSB particles (fig. S2a in Ugai *et al.*, 2007). Detection and quantification of mature protein X in our experiments was made almost impossible by the levels of arginine in protein X that, when digested by trypsin, will yield peptides too small to be detected by MS/MS. Comparison of levels of pre-X between WT and the dV/TSB virus is, however, further complicated because the point mutations in pre-X are present in the peptides detected by our MS/MS analysis. MS/MS-based quantitative analysis is only possible between samples with identical peptide sequences.

**Fig. 2.**
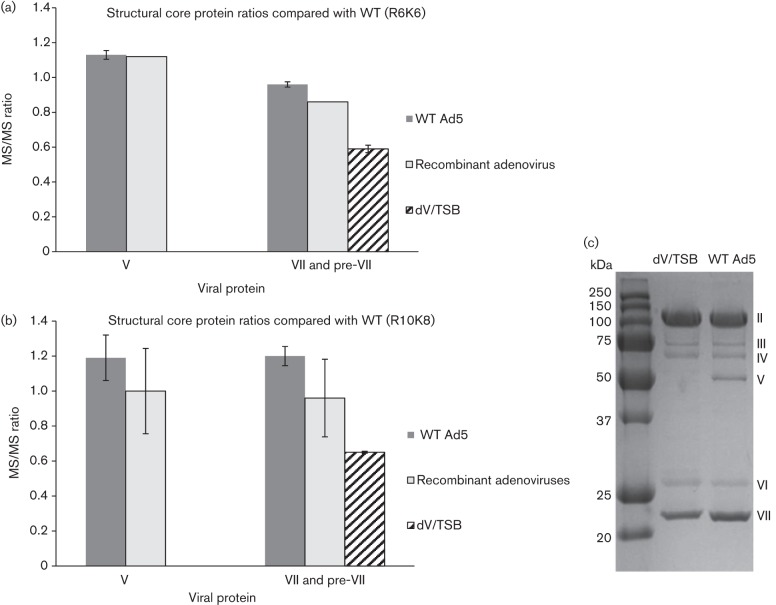
Analysis of the relative abundance of proteins V and VII in mature WT or dV/TSB particles. (a, b) The various batches of mature WT, recombinant adenovirus and dV/TSB particles were compared with a single batch of WT Ad5 labelled with R6K6 (a) or R10K8 (b). Both (a) and (b) show the data on the relative abundance of the virus core proteins V and pre-VII only. (c) Coomassie blue staining analysis of the mature WT Ad5 and dV/TSB is shown to allow a direct comparison of the abundance of viral proteins in each preparation.

We found no evidence that WT Ad5, the recombinant adenoviruses and dV/TSB have cellular proteins consistently associated with or packaged into virus particles. As we reliably detect and quantify viral proteins with a copy number of just two, our data make it less likely that there are cellular proteins deliberately packaged or associated with the virus particles. What is important to bear in mind, however, is that other cellular proteins or post-translationally modified proteins may not be detected by our analysis. In addition, it is also possible that specific combinations of cell line and preparation method may allow consistent association of a protein with the virus in that context only.

We have analysed the composition of WT, recombinant and dV/TSB particles using SILAC-based quantitative proteomics. To the best of our knowledge, this kind of analysis has only been used before to examine pseudorabies (Michael *et al.*, 2006). As in that study, the major and minor structural proteins could be readily identified and quantified, showing the expected ratios in both mature and immature particles. We detected a wide range of cellular proteins in these samples but not with consistent ratios of abundance that might indicate deliberate packaging or association with virus particles (e.g. as we have found for the major structural proteins of adenovirus). Our data show that dV/TSB virus has consistently reduced amounts of protein VII and there is no evidence that the missing protein V is replaced by a cellular protein. In the wider context, we propose that our approach is a valuable way to analyse the composition and batch-to-batch variation of WT, mutant and genetically engineered viruses, VLPs or nanoparticles.
